# Multi‐segment intussusception in infant with suspected celiac crisis: A case report

**DOI:** 10.1002/jpr3.70269

**Published:** 2026-11-05

**Authors:** Shambhawi Thakur, Angela Tran, Vivien Nguyen, Soo‐Jin Cho, Telly Cheung

**Affiliations:** ^1^ Department of Pediatrics University of California, San Francisco Oakland California USA; ^2^ Department of Pathology University of California, San Francisco San Francisco California USA

**Keywords:** celiac crisis, gluten‐free diet, intussusception, tissue transglutaminase

## Abstract

Celiac crisis (CC) is a rare, life‐threatening manifestation of often undiagnosed or inadequately treated celiac disease (CeD). In infants with nonclassical presentations of CeD, timely diagnosis can be challenging but critical to reducing end‐organ damage and serious complications, such as CC. We report a case of a 14‐month‐old infant presenting with 1 month of inappetence, malnutrition, and abdominal pain in the absence of diarrhea. The infant was found to have extraintestinal manifestations suggestive of CC, such as electrolyte derangement, hypoalbuminemia, and neurologic dysfunction. However, the infant also presented with unusual multi‐segment small bowel‐small bowel intussusception prior to the diagnosis of CeD. Intussusception may reflect severe small bowel enteropathy in CeD. Hypoalbuminemia and intestinal edema seen in CC may further precipitate small bowel‐small bowel intussusception. Future studies should clarify distinct phenotypes of infantile‐onset CeD associated with more severe CC.

## 1 INTRODUCTION

Celiac disease (CeD) is a chronic autoimmune enteropathy triggered by gluten consumption in genetically predisposed individuals. While the incidence of CeD is accelerating, diagnosing CeD in infants and toddlers remains challenging due to subclinical or nonclassical presentations.^1^ In rare cases, untreated CeD may precipitate celiac crisis (CC), a rare, life‐threatening complication, most commonly seen in children.^2^

Herein, we present a case of an infant evaluated for intussusception and suspected to have CC as a manifestation of undiagnosed CeD.

## 2 CASE REPORT

A 14‐month‐old previously healthy female with sickle cell trait presented to an emergency department (ED) with 1 month of inappetence and weight loss. Her symptoms progressed with marked abdominal pain, distension, lethargy, and vomiting in the absence of diarrhea. Although she had previously met her developmental milestones, she regressed and stopped walking independently. Abdominal ultrasound suggested intussusception versus volvulus, so she was transferred for surgical consultation.

On presentation to our ED, the patient was afebrile and hemodynamically stable. Her weight had declined over 4 months, falling from the 50th to 22nd percentile (*Z*‐score, 0.22 to −0.78). Her weight‐for‐length had decreased from the 39th to 13th percentile (*Z*‐score, −0.28 to −1.13). She appeared lethargic and underweight. Her abdomen was tympanitic without tenderness. Cutaneous exam revealed pallor and scattered erythematous papules across the cheeks, hands, elbows, and knees.

Review of the external abdominal ultrasound demonstrated a small bowel‐small bowel intussusception in the hypogastric region. However, no signs of intussusception were noted on two subsequent abdominal ultrasounds. Pediatric surgery and gastroenterology were consulted, and she was admitted for fluid resuscitation and management of growth faltering. The next day, she developed refeeding syndrome, requiring electrolyte repletion and thiamine supplementation.

During admission, diagnostic work‐up revealed elevated tissue transglutaminase immunoglobulin A (128 U/mL; upper limit of normal, 7 U/mL). Laboratory studies showed hypoalbuminemia (2.6 g/dL), hypophosphatemia (3.0 mg/dL), microcytic anemia (hemoglobin 11.5 g/dL, mean corpuscular volume 73, ferritin 58 ng/mL, iron 14 µg/dL, transferrin saturation 10%), low vitamin D‐25‐OH (9 ng/mL), and low zinc (56 mcg/dL). Stool studies showed elevated alpha‐1‐antitrypsin (1.13 mg/g) and fecal calprotectin (149 mcg/g). Abdominal X‐ray demonstrated air‐fluid levels, prompting a contrast‐enhanced abdominal computed tomography scan that revealed several short‐segment small bowel‐small bowel intussusceptions (Figure 1).

**Figure 1:**
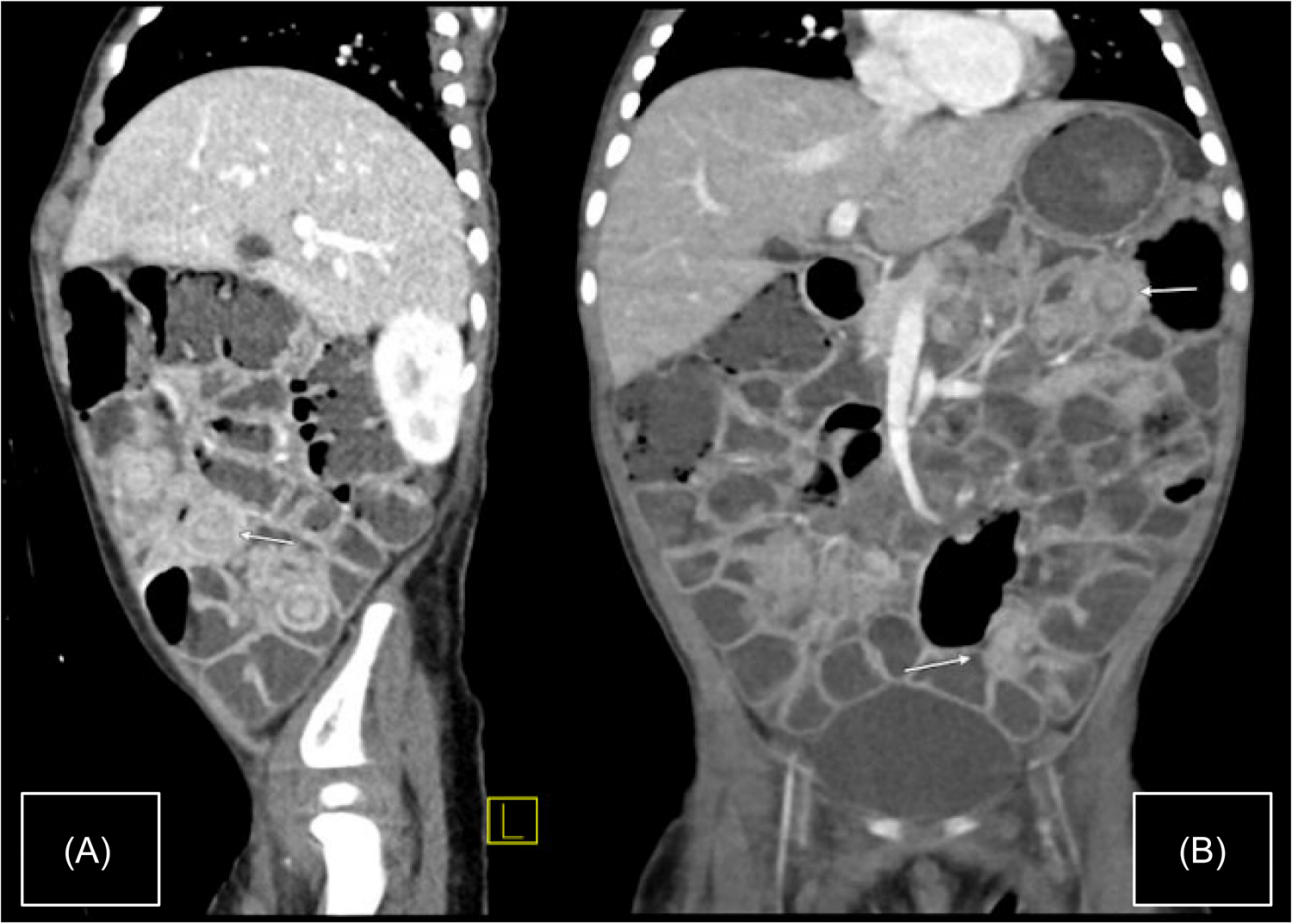
Abdominal computed tomography scan with contrast showing several short segment small bowel‐small bowel intussusceptions (indicated by the arrows) in coronal view (A) and sagittal view (B).

Endoscopy showed mucosal edema with flattened villi and scalloped folds in the duodenum (Figure 2). Histopathology of the duodenal bulb and distal duodenum revealed increased intraepithelial lymphocytes (>40 per 100 epithelial cells) and marked villus atrophy, confirming CeD with Marsh–Oberhuber 3B classification (Figure 3). The patient began a gluten‐free diet (GFD) with vitamin D supplementation. Nutrition was consulted to provide dietary education.

**Figure 2:**
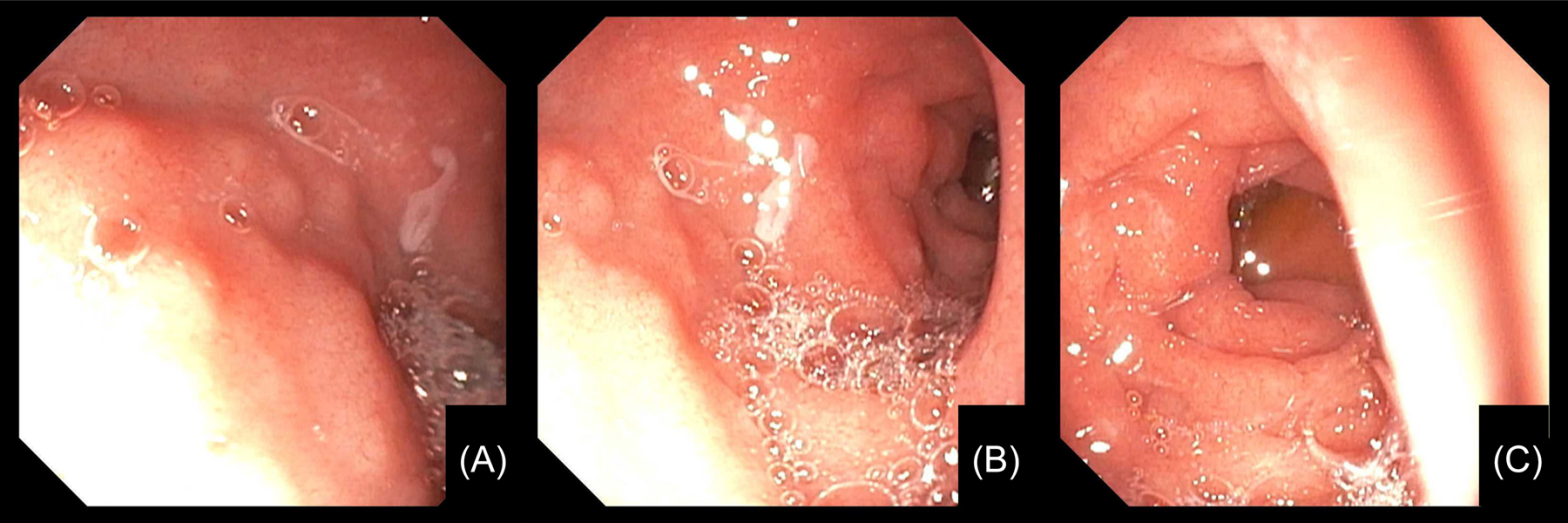
Esophagogastroduodenoscopy images showing mucosal edema with flattening and decreased mucosal folds as demonstrated by scalloped mucosa in the duodenal bulb (A, B) and the second portion of the patient's duodenum (C).

**Figure 3:**
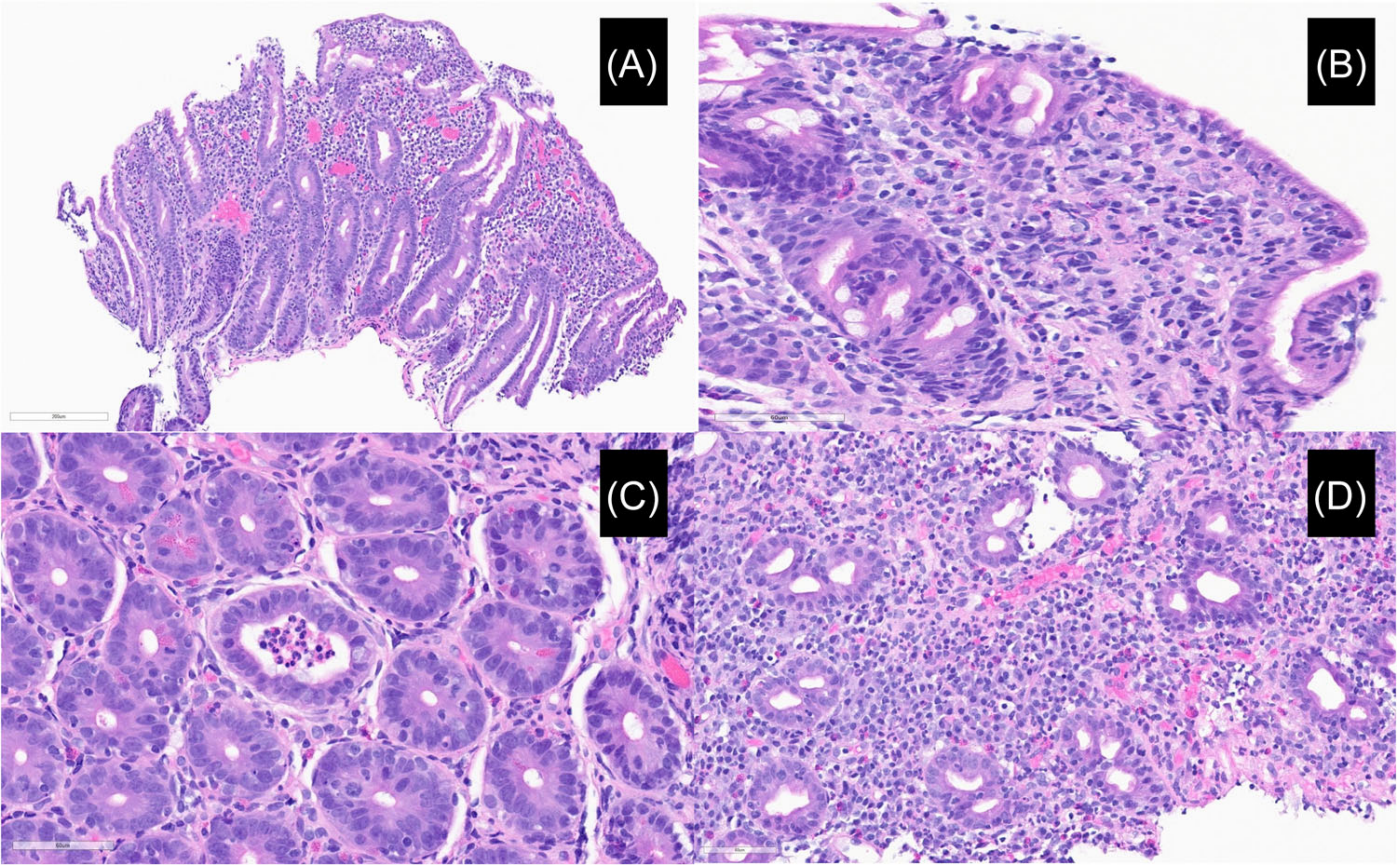
Histopathological examination of duodenum and duodenal bulb biopsy specimens using hematoxylin and eosin stain. Low‐magnification micrograph of the duodenal mucosa specimen (A) and high‐magnification micrograph of the duodenal bulb specimen (B) demonstrating increased intraepithelial lymphocytosis (> 40 per 100 epithelial cells) with marked villus atrophy, consistent with Marsh‐Oberhuber 3B. Micrographs of duodenum demonstrating foci of active inflammation with crypt abscess formation (C) and cryptitis with lamina propria neutrophilic inflammation (D).

By day three of hospitalization, the patient's symptoms, nutritional status, and neurological function had improved, and she was discharged. At 1‐month follow‐up with pediatric gastroenterology, her weight recovered to the 33rd percentile (*Z*‐score, −0.43), her weight‐for‐length improved to the 50th percentile (*Z*‐score, −0.01), and her rash had resolved.

## 3 DISCUSSION

CC is a life‐threatening complication that predominantly affects newly diagnosed CeD among children.^2^ While CC affects <1% of all individuals with CeD, recognizing the presentation is critical to diagnosis. Promptly initiating a GFD limits mortality risk. Current diagnostic guidelines for CC include gastrointestinal symptoms and ≥2 of the following: severe dehydration, renal dysfunction, neurological dysfunction, metabolic acidosis, electrolyte derangements, hypoproteinemia, and/or weight loss over 4.5 kg.^3^

Herein, we report the youngest known presentation of suspected CC in North America. A 14‐month‐old female infant had extraintestinal features of CC, including electrolyte derangement, hypoalbuminemia, malnutrition, and neurological dysfunction (i.e., lethargy and developmental regression). Studies indicate that most CC cases present with diarrhea (88%), abdominal pain (73%), vomiting (56%), electrolyte derangement (64%–90%), and hypoalbuminemia (77%) with few exhibiting neurologic symptoms (11%).^2^ Our patient's lack of diarrhea and non‐rapid progression of gastrointestinal symptoms would be atypical for CC under current diagnostic criteria.^3^ Although her presentation may suggest an alternative diagnosis, other cases demonstrate that CC may have predominantly extraintestinal features even in the absence of gastrointestinal symptoms.^4^ Revising current guidelines could broaden the clinical spectrum of CC, allow stratification by severity, and better account for potential comorbidities, such as refeeding syndrome and intussusception.^5^, ^6^, ^7^

We suspect that the severity of her CeD precipitated from prolonged mucosal injury, as evidenced by functional iron deficiency, zinc malabsorption, vitamin D deficiency, protein‐losing enteropathy, and malnutrition. While the diffuse papular rash may have represented dermatitis herpetiformis, resolution within 1 month after starting a GFD would be atypical.

Her initial diagnosis was complicated by transient multi‐segment small bowel‐small bowel intussusception that self‐resolved. Extensive small bowel enteropathy seen in severe CeD may create lead points that precipitate intussusception. Prior studies describe small bowel‐small bowel intussusception presenting in children with CeD, but remains rarely reported in CC. To the best of our knowledge, only one reported case in pediatrics demonstrated both intussusception and the extraintestinal manifestations observed in our patient with suspected CC.^7^ Intussusception may present in 25% of children with newly diagnosed CeD, representing a key diagnostic marker for CeD and potentially greater severity in cases of CC.^8^

Despite the known co‐occurrence between infections and CC, our patient lacked fever and diarrhea. Thus, no infectious workup was completed to identify enteric pathogens observed in CC (i.e., rotavirus and *Salmonella* species). Notably, acute vomiting, diarrhea, and weight loss—often attributed to infectious gastroenteritis—are frequent in CC and should raise clinical suspicion for CC. Consistent with studies showing improvement in ≥95% cases, our patient had symptom resolution within days and growth recovery within weeks of gluten elimination. While CC is potentially life‐threatening, <5% of cases require intensive care.^2^ However, poor recognition and under‐reporting of CC may limit estimates of true mortality risk.

## 4 CONCLUSION

This case highlights the broad and non‐gastrointestinal manifestations of suspected CC in infantile‐onset CeD. CC is a severe consequence of unrecognized CeD in children that should be considered during clinical assessment given the mortality risk. Intussusception in a child with growth failure, electrolyte derangements, and neurologic deterioration should prompt evaluation of CC to avoid fatal complications. Further studies are needed to clarify distinct phenotypes of infantile‐onset CeD linked to more severe CC.

## CONFLICT OF INTEREST STATEMENT

The authors declare no conflicts of interest.

## ETHICS STATEMENT

This study was conducted in accordance with the Declaration of Helsinki. Informed consent was obtained for the publication of the patient's medical information and photographs from the parent(s) of the patient with the understanding that this information may be publicly available. The informed consent was provided by the authors at the time of article submission to the journal stating that the patient's parent(s) gave consent for all of the patient's case details to be published in print and/or online.
